# The perceived effectiveness of MERS-CoV educational programs and knowledge transfer among primary healthcare workers: a cross-sectional survey

**DOI:** 10.1186/s12879-019-3898-2

**Published:** 2019-03-21

**Authors:** Meshal Aldohyan, Nedal Al-Rawashdeh, Farouk M. Sakr, Saeed Rahman, Ali I. Alfarhan, Mahmoud Salam

**Affiliations:** 1 Pharmaceutical care department -Ministry of National Guard Health Affairs, King Abdullah International Medical Research Center, King Saud bin Abdulaziz University for Health Sciences, Riyadh, Kingdom of Saudi Arabia; 2 Research office, King Abdullah International Medical Research Center, King Saud bin Abdulaziz University for Health Sciences, Ministry of National Guard Health Affairs, Riyadh, Kingdom of Saudi Arabia; 3 Office of Scientific Affairs and Research, King Hussein Cancer Center, Amman, Jordan; 4 Riyadh Colleges of Dentistry and Pharmacy, Riyadh, Kingdom of Saudi Arabia; 5 Department of Family Medicine & PHC-Ministry of National Guard Health Affairs, King Abdullah International Medical Research Center, King Saud bin Abdulaziz University for Health Sciences, Riyadh, Kingdom of Saudi Arabia; 6 Science and technology unit, King Abdullah International Medical Research Center, King Saud bin Abdulaziz University for Health Sciences, Ministry of National Guard Health Affairs, Riyadh, Kingdom of Saudi Arabia

**Keywords:** MERS, Coronavirus, Knowledge transfer, Education, Primary health

## Abstract

**Background:**

Knowledge transfer of Middle East respiratory syndrome coronavirus (MERS-CoV) involves the dissemination of created/acquired information on MERS-CoV in hospitals, making this information accessible to all healthcare workers (HCWs). This study evaluated the perceived effectiveness of MERS-CoV educational programs and knowledge transfer among primary care HCWs at a hospital in Saudi Arabia that witnessed the largest outbreak of confirmed MERS-CoV cases in this country.

**Methods:**

A survey was distributed among primary care HCWs at five clinics in Saudi Arabia in 2016. Those with non-direct patient care responsibilities were excluded. Their knowledge was evaluated against facts published by Mayo Clinic Foundation, and its percentage mean score (PMS) ± standard deviation was calculated. HCWs’ perceived effectiveness of educational programs and knowledge transfer was classified as negative or positive.

**Results:**

Sample comprised of 404 HCWs, of which 64% were females and 36% were males. Almost 26% were ≤ 30 years old, and 42% had > 10 years of work experience. Almost 46.5% were nurses, 23.0% physicians, 18.1% were pharmacists, and 12.4% were technical staff. PMS for knowledge was 71.1 ± 19.4. The prevalence of negative perceptions towards educational programs was 22.5% and of knowledge transfer was 20.8%. Older(> 40 years of age) and more experienced(> 10 years) HCWs had the highest PMS for knowledge(73.4 ± 18.9,*P* = 0.005 and 76.9 ± 15.7,*P* < 0.001 respectively). Negative perceptions of educational programs (49.4 ± 20.7; *P* < 0.001) and knowledge transfer (46.0 ± 19.7; *P* = 0.001) were associated with a lower knowledge PMS. Males were 2.4[95% confidence interval 1.4–4.2] times and 2.0[1.1–3.5] times more likely to have negative perceptions of educational programs and knowledge transfer (adjusted (adj.)*P* = 0.001 and adj. *P* = 0.023, respectively). Physicians/pharmacists were 1.8[1.03–3.11] and 2.8[1.6–5.0] times more likely to have negative perceptions of both outcomes (adj. *P* = 0.038 and adj. *P* = 0.001, respectively). Less experienced HCWs were 2.1[1.3–3.5] times and 4.9[2.6–9.2] times more likely to exhibit negative perceptions of the two outcomes (adj. *P* < 0.001 each).

**Conclusions:**

A negative perception of the effectiveness of MERS-CoV knowledge transfer was associated with poorer knowledge and was more prevalent among male HCWs, physicians/pharmacists and less experienced HCWs. Hospitals should always refer to efficient knowledge sharing and educational strategies that render beneficial outcomes to patients, HCWs, and the public community.

**Electronic supplementary material:**

The online version of this article (10.1186/s12879-019-3898-2) contains supplementary material, which is available to authorized users.

## Background

Middle East respiratory syndrome coronavirus (MERS-CoV) has created an epidemiological and clinical crisis within 27 countries in North Africa, Europe, Asia and the USA but mainly in the Middle East (Kingdom of Saudi Arabia) [1]. It is a viral respiratory illness, initially discovered in 2012 and speculated to have originated from camels or bats in Saudi Arabia, with subsequent spread to humans and across borders [2]. Since 2012, a minimum of 1415 laboratory-confirmed cases have been reported in Saudi Arabia, of which 610 patients have died, 833 have recovered and two were under treatment [3]. High-risk groups were those in close contact with camels, geriatric persons, pregnant women, healthcare workers (HCWs) and those with pre-existing comorbidities [1]. MERS-CoV infection ranged from asymptomatic or mild respiratory symptoms to severe acute respiratory disease and even death, which was reported in three to four out of every 10 reported MERS-CoV cases [4]. Biologic samples of subjects with a suspected MERS-CoV infection (based on clinical symptoms) and of those exposed to reported MERS-CoV cases are tested using real-time reverse transcription polymerase chain reaction (rRT-PCR) assays. Serology, such as an enzyme-linked immunosorbent assay and immunofluorescence assay, is also used to confirm MERS-CoV by the presence of antibodies [5].

In Saudi Arabia, a series of modifications was applied to the patient pathways while visiting the emergency departments or admitted as in-patients. This included segregating patients during triage based on prioritizing the inflow of patients by their chief complaints, bed availability and screening of flu symptoms/history of exposure. The infrastructure of the medical facility, particularly the ventilation system and isolation capacity of rooms, was subject to changes. Some hospital wards and staff (especially nurses) were dedicated specifically to confirmed MERS-CoV cases to limit the chance of cross-contamination across wards and HCWs. The Infection Prevention and Control Department (IPCD) at SMNG-HA, in particular, was on high alert for such MERS-CoV outbreaks, especially with the evident transmission of viral infections between patients and HCWs at SMNG-HA. Crisis management required a rapid implementation of adequate infection prevention, control procedures and case isolation, in addition to collaboration and coordination with local and international consultations.

Exceptional efforts have been made by the IPCD to apply the latest and most effective means of universal standard precautions throughout the MERS-CoV crisis. Rules and regulations pertinent to infection control and prevention have been revisited and environmental surveillance has been carried out regularly to ensure that all wards are equipped with suitable protection and precautionary gear. Numerous seminars, workshops and awareness campaigns have been launched for HCWs of all disciplines to boost their knowledge on MERS-CoV, as well as their morale, to maintain a high-quality, safe and dedicated service for the patients. The latest updates issued on MERS-CoV from the World Health Organization (WHO), the Centers for Disease Control and Prevention (CDC), collaborative task forces (local and regional) and researchers have been circulated regularly among all HCWs and across all managerial levels.

Numerous research studies have been conducted and published on the perception, knowledge and attitude of HCWs towards MERS-CoV. It is rare to find a HCW who has not attended an educational program on MERS-CoV in Saudi Arabia. Dissemination of MERS-CoV information/updates or knowledge transfer within a healthcare organization is a process in which this information is created, generated or acquired, and then organized and distributed within the system to ensure it is accessible to all HCWs. One of the mechanisms of knowledge transfer is personalization whereby knowledge is transferred from one individual to another, while the other is codification where knowledge is converted into products such as documents, images and videos [6]. The need to transfer efficiently the precautionary regulations and updates about MERS-CoV to large numbers of HCWs necessitates the mechanism of codification [7]. In addition, knowledge transfer or information sharing was found to be positively associated with job satisfaction [8].

Authors hypothesized that although the dissemination of knowledge and updates on MERS-CoV among HCWs has been given full consideration, these HCWs might have reservations on the effectiveness and quality of MERS-CoV related educational offerings. Therefore, there was an emerging need to evaluate the perceived effectiveness of MERS-CoV educational programs and knowledge transfer from the HCW’s perspective, in a setting that witnessed the largest outbreak of confirmed MERS-CoV cases in Saudi Arabia.

## Methods

This was a cross-sectional study, based on an anonymous survey in English distributed among HCWs at the primary healthcare centers in SMNG-HA medical centers, in Riyadh, Saudi Arabia, between October and December 2016. The SMNG-HA is the second-largest healthcare sector in the country, second only to the Saudi Ministry of Health, and provides healthcare services to the community of national guards, their dependents and employees [9]. The targeted primary healthcare centers were five randomly selected clinics out of 11 clinics that employ physicians, pharmacists, technicians and nurses. These clinics serve a population of 60,000 registrants, with a rate of four visits per registrant annually. Eligible primary care HCWs were targeted as being in frontline contact with potential confirmed cases of MERS-CoV. Those occupying positions of management, education or non-direct patient care were excluded. Medical and nursing students were also excluded. This study was ethically approved by the Institutional Review Board at King Abdullah International Medical Research Center, King Saud bin Abdul-Aziz University for Health Sciences, SP-16/191.

The provisioned sample size in this study was calculated based on a reported level of knowledge between 43.3 and 57.4% by Alkot et al. among HCWs in the Western region of Saudi Arabia. Assuming an expected level of knowledge of 57%, with a 95% confidence limit (z = 1.96), and a margin of error 5%, the estimated sample size for this study was 376. For convenience, all eligible HCWs at the targeted setting were invited to participate in this survey, to overcome a 25% non-response rate. The survey was provided in a sealed envelope with a cover letter that described the objectives of the study. The survey was in English language, as the targeted study participants were English literate and the educational offerings provided at the targeted setting were also in English. Participants who agreed to enroll in this study hand-signed an agreement statement at the end of an informed consent, with no need for any personal identifier. The data collection tool comprised the characteristics of the HCW, principally gender, age category (years), job position and experience (years) [10]. The knowledge of HCWs was measured using 16 statements based on undisputed facts published in the literature and issued by the Mayo Foundation for Medical Education and Research in 2018 [11]. Correct answers were scored “1”, while wrong/don’t know answers were scored “0”. The percentage mean score (PMS) of knowledge was calculated by adding the correct responses of the 16 statements, dividing the score by 16 and multiplying it by 100 (range of score 0 to 100). The perceived effectiveness of the MERS-CoV educational programs was measured using one statement: “Prevalence of MERS can be reduced by active participation of healthcare workers in the hospital infection control program”, while the perception of knowledge transfer was measured by one statement: “Any related information about MERS should be disseminated among healthcare workers”. Both statements were rated on a four-point Likert scale (strongly disagree, disagree, agree and strongly agree). Those who responded by disagree or strongly disagree were classified as having a negative perception, while those who responded agree or strongly agree were classified as having a positive perception. The negative perception rate was calculated by dividing the number of participants who had negative responses over the total number of respondents. In addition, participants were asked about the source of MERS-CoV information. The survey was piloted on a group of five HCWs, and their subjective comments were considered. The internal consistency or reliability (Cronbach’s alpha) of the knowledge domain measured 0.67 (Additional file 1).

Data were analyzed using the Statistical Package for Social Studies (SPSS 25; IBM Corp., New York, NY, USA). HCW characteristics, perceptions (negative vs positive) and incorrect knowledge response statements were presented in terms of frequencies and percentages, while the PMS of knowledge was presented as the mean ± standard deviation. Missing data were replaced by the average of the total, and outliers were dropped out. Pearson’s chi-square test was used to test categorical outcomes across HCW characteristics, while a Mann–Whitney test and a Kruskal–Wallis test were used to test the non-normally distributed PMS of knowledge scores. Two binary logistic regression models were constructed to determine the factors significantly associated with negatively perceived effectiveness of MERS-CoV educational programs and knowledge transfer. Due to the small subgroup size of job positions, nurses were grouped with technicians, while pharmacists were grouped with physicians. These two subgroups had job positions comparable in terms of the educational levels, scope of practice and nature of patient care. The adjusted odds ratios [95% confidence interval] were calculated, and statistical significance was set at a value of *P* < 0.05.

## Results

### Sample and outcome characteristics

Initially, 600 surveys were distributed among HCWs; 404 participants agreed to enroll and completed the survey (response rate 67.3%). Those who did not participate were mainly either off duty or busy with their workload. Females constituted 64.1% of the sample, while males comprised 35.9%. Almost 26% were ≤ 30 years old, 29% were 30–40 years old and 45% were > 40 years old. The majority (46.5%) were nurses, followed by physicians (23.0%), pharmacists (18.1%) and technical staff (12.4%). Most HCWs (42.1%) had accumulated > 10 years of work experience, with 29.2% having < 5 years of experience and 28.7% having 5–10 years (Table 1). Overall, 36.1% of respondents claimed that their main source of information was the internet, while 47.8% reported more than one source, including research studies, books, media and others.

**Table 1:** Health care workers’ and outcomes’ characteristics

	n(%)
Gender	
Male	145 (35.9%)
Female	259 (64.1%)
Age (years)	
≤ 30	107 (26.5%)
30–40	116 (28.7%)
> 40	181 (44.8%)
Job title	
Physician	93 (23.0%)
Nurse	188 (46.5%)
Pharmacist	73 (18.1%)
Technical	50 (12.4%)
Experience (years)	
< 5	118 (29.2%)
5–10	116 (28.7%)
> 10	170 (42.1%)
Percentage Mean Score of Knowledge	
(Mean ± SD)	71.1 ± 19.4
Perception towards effectiveness of MERS CoV educational programs	
Negative	91 (22.5%)
Positive	313 (77.5%)
Perception towards knowledge transfer of MERS CoV updates	
Negative	84 (20.8%)
Positive	320 (79.2%)

*n* frequency, *%* percentage, *SD* standard deviation

The PMS of knowledge score was 71.1 ± 19.4. The most common incorrect response to the statements (83.4%) was that for “incubation time for virus”, followed by 43.6% with an incorrect response to the statement that “antibiotics are the first-line treatment for the management of MERS-CoV”. Other incorrect responses to statements are listed in chronological order in Table 2. Overall, 22.5% of participants reported a negative perceived effectiveness of MERS-CoV educational programs, while 20.8% had a negative perception of knowledge transfer.

**Table 2:** Frequency of wrong responses to individual knowledge statements

		n(%)
1	Incubation time for virus is 14–28 days	337 (83.4%)
2	Antibiotics are the first line treatment for the management of MERS-CoV	176 (43.6%)
3	Polymerase chain reaction can be used to diagnose MERS-CoV	163 (40.3%)
4	Washing hands vigorously (soap/water) for 20 s helps in prevention/transmission of disease	130 (32.2%)
5	Vaccination of MERS-CoV is available in market	98 (24.3%)
6	MERS-CoV is caused by alpha coronavirus	95 (23.5%)
7	The main source of MERS virus is plant	86 (21.3%)
8	Transmission of MERS-CoV infection can be prevented by using universal precautions.	82 (20.3%)
9	MERS patients should be kept in isolation	78 (19.3%)
10	Gowns, gloves, mask and goggles must be used when dealing with MERS patients	75 (18.6%)
11	People with co-morbidities are more likely to be infected	73 (18.1%)
12	Special caution must be taken if a person presents with MERS symptoms from the Arabian Peninsula	72 (17.8%)
13	MERS-CoV patients develop severe acute respiratory illness	61 (15.1%)
14	MERS-CoV spreads through close contact like caring and/or living with infected persons	54 (13.4%)
15	Fever, cough and shortage of breath are hallmark symptoms of MERS-CoV	47 (11.6%)
16	MERS-CoV can be fatal	47 (11.6%)

*n* frequency, *%* percentage

### Factors associated with outcomes

With regard to the perceived effectiveness of MERS-CoV educational programs, male HCWs had significantly a more negative perception than female HCWs (*n* = 52, 35.9%, vs *n* = 39, 15.1%, respectively; *P* < 0.001). Pharmacists (*n* = 24, 32.9%) and physicians (*n* = 30, 32.3%) reported more negative perceptions than technical staff (*n* = 9, 18.0%) and nurses (*n* = 28, 14.9%) (*P* = 0.001). HCWs with work experience of < 5 years had the most negative perceptions in comparison with the other groups (*P* = 0.001). A number of factors were associated with a negative perception of knowledge transfer of MERS-CoV information. Male HCWs had a greater negative perception than females (*n* = 47, 32.4%, vs *n* = 37, 14.3%, respectively; *P* < 0.001). Physicians (*n* = 31, 33.3%) and pharmacists (*n* = 23, 31.5%) had more negative perceptions of knowledge transfer in comparison with technical staff (*n* = 9, 18.0%) and nurses (*n* = 21, 11.2%) (*P* < 0.001). Junior HCWs with work experience of < 5 years (35.4%) had the highest rate of negative perception of knowledge transfer (*P* < 0.001) (Table 3). HCWs > 40 years old (PMS 73.4 ± 18.9) had the highest knowledge scores in comparison with the other age groups (*P* = 0.005). More experienced HCWs (> 10 years) also had the highest knowledge scores (PMS 76.9 ± 15.7; *P* < 0.001). Those who had a negative perception of the effectiveness of MERS-CoV educational programs (PMS 49.4 ± 20.7) and of knowledge transfer of MERS-CoV updates (PMS 46.0 ± 19.7) both had lower knowledge scores in comparison with the positive-perception group (*P* < 0.001 and *P* = 0.001, respectively), Table 4.

**Table 3:** Perceived effectiveness of MERS-CoV educational programs and its knowledge transfer across particpant characteristics

	Perceived effectiveness of MERS CoV educational programs		Perception towards knowledge transfer of MERS CoV updates	
	Negative n(%)	Positive n(%)	Negative n(%)	Positive n(%)
Gender				
Male	52 (35.9%)	93 (64.1%)	47 (32.4%)	98 (67.6%)
Female	39 (15.1%)	220 (84.9%)	37 (14.3%)	222 (85.7%)
	χ2 = 23.05, *P* < 0.001*		χ2 = 18.55,*P* < 0.001*	
Age (years)				
≤ 30	30 (28.0%)	77 (72.0%)	28 (26.2%)	79 (73.8%)
30–40	18 (15.5%)	98 (84.5%)	23 (19.8%)	93 (80.2%)
> 40	43 (23.8%)	138 (76.2%)	33 (18.2%)	148 (81.8%)
	χ2 = 5.285, *P* = 0.071		χ2 = 2.664, *P* = 0.264	
Job title				
Physician	30 (32.3%)	63 (67.7%)	31 (33.3%)	62 (66.7%)
Nurse	28 (14.9%)	160 (85.1%)	21 (11.2%)	167 (88.8%)
Pharmacist	24 (32.9%)	49 (67.1%)	23 (31.5%)	50 (68.5%)
Technical	9 (18.0%)	41 (82.0%)	9 (18.0%)	41 (82.0%)
	χ2 = 16.392, *P* = 0.001*		χ2 = 24.776, *P* < 0.001*	
Experience (years)				
< 5	40 (33.9%)	78 (66.1%)	42 (35.4%)	76 (64.6%)
5–10	24 (20.7%)	92 (79.3%)	27 (23.3%)	89 (76.7%)
> 10	27 (15.9%)	143 (84.1%)	15 (8.8%)	155 (91.2%)
	χ2 = 13.269, *P* = 0.001*		χ2 = 30.918, *P* < 0.001*	

*n* frequency, *%* percentage, *χ2* Chi-square, *P P*-value, *: significance at < 0.05

**Table 4:** Knowledge scores across various sample characteristics

	PMS Knowledge Mean ± SD
Gender	
Male	67.3 ± 23.4
Female	73.3 ± 16.5
	Z = -1.589, *P* = 0.112
Age (years)	
≤ 30	66.4 ± 20.7
30–40	72.0 ± 18.4
> 40	73.4 ± 18.9
	χ^kwt^ = 10.51, df = 2, *P* = 0.005*
Job title	
Physician	69.5 ± 24.2
Nurse	74.3 ± 14.0
Pharmacist	64.9 ± 23.2
Technical	71.3 ± 19.0
	χ^kwt^ = 5.644, df = 3, *P* = 0.130
Experience (years)	
< 5	63.9 ± 22.5
5–10	70.0 ± 18.4
> 10	76.9 ± 15.7
	χ^kwt^ = 28.39, df = 2, *P* < 0.001*
Perception towards effectiveness of MERS CoV educational programs	
Negative	49.4 ± 20.7
Positive	77.3 ± 13.9
	Z = -10.43, *P* < 0.001*
Perception towards knowledge transfer of MERS CoV updates	
Negative	46.0 ± 19.7
Positive	77.5 ± 13.2
	Z = -11.248, *P* = 0.001*

*n* frequency, *%* percentage, *Z* Mann Whitney test, *χ*^*kwt*^ Kruskal–Wallis test, *df* degree of freedom, *P P*-value, *: significance at < 0.05

Logistic regression analyses showed that male HCWs were 2.4 [1.4–4.2] times and 2.0 [1.1–3.5] times more likely to have negative perceptions of the effectiveness of MERS-CoV educational programs and knowledge transfer in comparison with female HCWs (adj. *P* = 0.001 and adj. *P* = 0.023, respectively). Physicians and pharmacists combined were also 1.8 [1.03–3.11] and 2.8 [1.6–5.0] more likely to have negative perceptions of the effectiveness of MERS-CoV educational programs and knowledge transfer, respectively, than nurses/technical staff (adj. *P* = 0.038 and adj. *P* = 0.001, respectively). Less experienced HCWs with ≤10 years of work experience were 2.1 [1.3–3.5] times and 4.9 [2.6–9.2] times more likely to exhibit negative perceptions of the two outcomes in comparison with more experienced HCWs with > 10 years of experience (adj. *P* < 0.001 each), Table 5.

**Table 5:** Significantly associated factors with negative perceptions towards MERS CoV educational programs and knowledge transfer

	Negative perception towards effectiveness of MERS CoV educational programs			Negative perception towards knowledge transfer of Mers CoV updates		
	β (S.E.)	Adj.*P*-value	Adj.OR[95% CI]	β (S.E.)	Adj.P-value	Adj.OR[95% CI]
Sex Female = 0; Male = 1	0.89 (0.28)	0.001*	2.4 [1.4–4.2]	0.67 (0.29)	0.023*	2.0 [1.1–3.5]
Specialty of physician Nurse/Technicians = 0 Physicians/pharmacists = 1	0.58 (0.28)	0.038*	1.8 [1.03–3.11]	1.02 (0.30)	0.001*	2.8 [1.6–5.0]
Experience (years) > 10 = 0; ≤10 = 1	0.74 (0.27)	0.005*	2.1 [1.3–3.5]	1.6 (0.32)	< 0.001*	4.9 [2.6–9.2]
Constant	−2.36 (0.27)	< 0.001*	0.094	−3.22 (0.35)	< 0.001*	0.040

*β* coefficient of determination, *S.E*. standard error, *adj*. adjusted, *OR* odds ratio, *CI* confidence interval, *P P*-value, *: significance at < 0.05

## Discussion

MERS-CoV educational programs at healthcare institutions are a formal and reliable channel to deliver essential knowledge to HCWs. For the sake of personal safety, job satisfaction and work morale, HCWs should not pass up any opportunity to increase their theoretical knowledge and practical skills. Hospital administrators do not necessarily face the challenge of producing new information, as an immense amount of valuable information already exists in the literature. The problem arises from the fact that current knowledge is either poorly structured or inaccessible to HCWs [12]. For example, advanced practice nurses are observed to be “knowledge brokers” in a sense that they act as disseminators of knowledge among the nursing body. Furthermore, health educators retrieve different types of evidence, synthesize it in different forms, translate it by evaluation, interpret it and then distribute it among nurses [13]. Health education can improve levels of awareness and perception among HCWs towards MERS-CoV infections [14], and these higher levels of knowledge can aid in the control of disease outbreaks [15]. However, published evidence in Saudi Arabia has shown that there is limited knowledge on MERS-CoV (both microbiological and virological aspects) among HCWs in southern Saudi Arabia [16]. Another study also claimed that knowledge about emerging infectious diseases was poor, and that infection control practices were suboptimal and also seemed to be overestimated [17]. The association between younger age and less experience on one hand and lower knowledge scores on the other was a reasonable finding. Similar to literature findings, the knowledge of HCWs in this setting was suboptimal and gaps remain that should direct the focus towards the mechanisms and quality of knowledge transfer.

Dissemination of MERS-CoV updates using e-mail, the internet, institutional announcements, employee meetings, the media and even personal communications are all methods of knowledge transfer. HCWs can experience knowledge transfer both passively, absorbing information unconsciously, and actively. Investigators in this study were curious to know how HCWs perceived the transfer of knowledge about MERS-CoV and why this would be of concern to hospital administrators. For instance, knowledge transfer has been adopted with regard to smoking as a health hazard, HIV transmission as a sexual risk and seat belts in motor vehicles as a safety measure. People are exposed almost daily to precautionary advice by a variety of methods but unfortunately still undertake high-risk activities and are exposed to these hazards. This occurs regardless of the duration, frequency and quality of awareness campaigns. Therefore, it is an aggravating concern that the repetitive exposure of HCWs to MERS-CoV campaigns might have created some sort of “tolerance”. HCWs might disremember or take lightly the acquisition of current or new updates about MERS-CoV precautions due to routine attendance of educational programs or repetitive circulation of e-mails.

Knowledge and skills must be passed on in a systematic way from expert to novice employees in a way that makes sense [18]. Managers who support work-empowering environments are actually boosting the engagement of participations in terms of knowledge transfer [19]. In fact, one of the key elements in seeking accreditation or managing crises such as MERS-CoV is knowledge communication, in the sense that effective communication ensures a purposeful exchange of information, thus allowing a more thorough understanding of the outbreak [20, 21]. Interactive workshops remain highly recommended for the sharing and transferring of knowledge among HCWs. However, one study noted that, although those who attended such workshops valued the expert input and discussions, after few months their sustainability of attendance was lost [22]. Some barriers to MERS-CoV knowledge transfer could be the inability of HCWs to recognize and articulate the instructions, personal opposition or resistance to change, the quality of the communication technologies, the absence of visual representations, language and cultural differences, deficiency in expertise, the work environment, a lack of job incentive/motivation, the organizational culture and others [23, 24].

Current efforts to manage the MERS-CoV crisis are directed towards developing educational programs that target both the community and HCWs [25]. A negative perception of MERS-CoV educational programs in this setting might result in outdated knowledge among HCWs, which jeopardizes their compliance with disease precautionary and control measures. A MERS-CoV task force committee pointed out that the Saudi Arabian Ministry of Health has posted updates on MERS-CoV through videos, posters, handouts, posters and an official website. Resilience against MERS-CoV increases with enriched education and awareness [25]. A Saudi Arabian study reported that HCWs were unaware of the availability of MERS-CoV information at their work areas; they did not feel they had sufficient training and were not confident about infection control guidelines. These factors may also contribute to having a negative perception of MERS-CoV-related educational programs [26]. One study reported that the interest in following disease updates among HCWs improved significantly after the implementation of a MERS-CoV educational program [14]. These programs improved the attitude of the HCWs towards governmental measures taken regarding the crisis [14]. HCWs often grasp their MERS-CoV educational information primarily from watching TV reports, or from the internet. A negative perception of knowledge transfer might be due to a pre-existing lack of trust in the media or in websites that might, to some degree, lack scientific credibility in comparison with educational programs provided in healthcare centers [27].

Knowledge itself is complex, and its transfer process within healthcare institutions carries many challenges [28]. One way to overcome these challenges is to determine the characteristics of HCWs who might be more likely to exhibit negative perceptions of knowledge transfer for significant MERS-CoV updates. In the literature, knowledge transfer has been investigated more frequently in manufacturing industries and firms, or among the public community. It has been seldom evaluated among HCWS [29], and never in a Middle Eastern setting or related to a MERS-CoV outbreak. A cross-national study suggested that organizational culture was a significant influence on the capacity of HCWs to engage in knowledge transfer [30]. A systematic review paper study stated that knowledge transfer could streamline productivity and coordinate the use of resources more efficiently [29]. This review paper claimed to be the first to review published research focused on the perceptions of HCWs about knowledge management [29]. Knowledge management was defined as having an efficient idea or new practice accepted and adopted by an individual or a group through communication channels (successful diffusion of ideas) [31]. This definition also applies to the dissemination of updated regulations on the outbreak of MERS-CoV. This information, once absorbed by people, should be sustained for as long as it is useful, and not decay over time [32]. Accordingly, a negative perception of the importance of knowledge transfer could be a warning sign of an interruption in this sustainability of retained information about MERS-CoV. Signs of information decay were evident among HCWs in this study, as those who had negative perceptions had lower knowledge scores about MERS-CoV in comparison with those who had positive perceptions.

One of the key goals of knowledge transfer is to educate and train the less experienced and/or the newly hired HCWs [33]. This phase of staff development is crucial yet stressful for novice HCWs, who are expected to acquire skills and competencies rapidly to ensure that a safe and quality service flow is maintained at the institution. This explains why HCWs with less work experience (< 5 years) had significantly more negative perceptions of knowledge transfer and the perceived effectiveness of MERS-CoV educational programs. As they gain more work experience, this perception improves as they realize the importance of education not only for their patients but also for their career advancement.

## Conclusions

The level of knowledge on MERS-CoV among HCWs in primary healthcare clinics in this setting was found to be less than optimal. As the frontline in the battle of disease prevention and control, HCWs are expected to be equipped with the relevant theoretical updates about MERS-CoV. Special consideration should be paid to younger and less experienced HCWs whose knowledge on MERS-CoV was moderately low. A negative perception of the knowledge transfer of MERS-CoV information and educational programs was associated with poorer knowledge. This negative perception was more prevalent among male HCWs, physicians/pharmacists and less experienced HCWs.

This study has been conducted at one setting, yet the struggle against MERS-CoV has not ended and will continue against future emerging strains of viruses and bacteria causing communicable diseases in other settings too. Knowledge is a valuable asset, and its holders within any healthcare institution should be retained and motivated so that they continue to spread their benefit among other HCWs. All healthcare institutions should always identify and refer to reliable sources of knowledge. For instance, the center for disease control and prevention is a leading national public health institute and accountable for disseminating up-to-dates on various infectious topics. In Saudi Arabia, the ministry for public health has designated communication channels to release updates on MERS-CoV on their websites, through scientific arenas, memorandums and helpdesks.

Knowledge sharing and management strategies in the healthcare sector can render beneficial outcomes to patients, HCWs, the organization and the public community [29]. In addition to the attendance of seminars or workshops, other methods of knowledge dissemination might involve launching of journal clubs among HCWs to discuss updates on MERS CoV. Audiovisuals at hospitals, such as educational videos on TV screens in lobbies or corridors, constantly enlighten HCWs. Deeper understanding of the negativity in the perception towards the quality or method of knowledge transfer necessitates a qualitative methodological approach, as face to face interviews with HCWs aid in determining the underlying reasons and at a more personal level. Furthermore, the execution of these strategies needs to be routinely monitored and evaluated so that the transfer of knowledge is time efficient and effective. Optimal theoretical knowledge and practical competence are two main indicators of successful knowledge transfer among HCWs. Last but not least, a number of key points can be noted:

- Middle East respiratory syndrome coronavirus (MERS-CoV) has created an epidemiological and clinical crisis within 27 countries around the globe.
- Exceptional efforts have been made by the Infection Prevention and Control Departments to apply the latest and most effective means of universal standard precautions.
- Numerous seminars, workshops and awareness campaigns have been launched to boost the knowledge on MERS-CoV among health workers, as well as their morale, to maintain a high-quality, safe and dedicated service for patients.
- The perceived effectiveness of MERS-CoV educational programs and knowledge transfer among health workers in this high risk setting was evaluated.
- Primary health workers were expected to be aware of the most recent updates on MERS-CoV, yet younger and less experienced HCWs had moderate knowledge.
- A negative perception of the effectiveness of MERS-CoV knowledge transfer was associated with poorer knowledge, and was more prevalent among male HCWs, physicians/pharmacists and less experienced HCWs.

## Additional file

Additional file 1: Questionnaire. (DOCX 136 kb)

## Abbreviations

adj.: Adjusted
CDC: Centers for Disease Control and Prevention
HCWs: Healthcare workers
IPCD: Infection Prevention and Control Department
MERS-CoV: Middle East respiratory syndrome coronavirus
PMS: Percentage mean score
rRT-PCR: Real-time reverse transcription polymerase chain reaction
SMNG-HA: Saudi Ministry of the National Guard Health Affairs
SPSS: Statistical Package for Social Studies
WHO: World Health Organization
