# Supplementary material for: The perceived effectiveness of MERS-CoV educational programs and knowledge transfer among primary healthcare workers: a cross-sectional survey
Source: BMC Infect Dis. 2019 Mar 21;19:273. doi: 10.1186/s12879-019-3898-2 (PMC6427879; doi:10.1186/s12879-019-3898-2)
Supplement: Supplementary file 1 — Questionnaire. (DOCX 136 kb) [file 12879_2019_3898_MOESM1_ESM.docx]

| Kingdom of Saudi Arabia  Ministry of National Guard Heath Affairs  King Abdul Aziz Medical City  Department of Family Medicine and PHC  **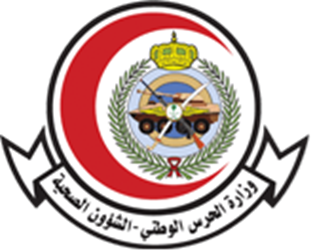**  **The perceived effectiveness of MERS-CoV educational programs and knowledge transfer among primary healthcare workers: a cross-sectional survey**  Dear participant,  As part of my partial fulfillment of my Masters in clinical pharmacy and pharm D, I would like to invite you to participate in a quick survey about the perceived effectiveness of MERS-CoV educational programs and knowledge transfer.  The survey is designed for research purposes only, thus no personal identifiers are required and your feedback will be dealt with utmost confidentiality.  For further details please contact us at: [almughaiseb@ngha.med.sa](mailto:ALMUGHAISEB@NGHA.MED.SA)  Best Regards |
| --- |

Kindly respond to the following questions

1**-** Gender?

- Male
- Female

2- Age?

…………………..

3- Job title?

- Physician
- Nurse
- Pharmacist
- Technical

4- Years of experience?

…………………..

4-Location of your clinic?:

- King Abdulaziz City (Iskan Clinics)
- King Saud City (Dirab PHC)
- NGCSC(UM ALHAMMAM)
- HCSC(KHASHMALAAN)
- KKMAH
- EMPLOYEE HEALTH CLINIC (EMH)
- RAFHA

Kindly respond to the following statements to the best of knowledge

| **Don't Know** | **No** | **Yes** |  |
| --- | --- | --- | --- |
|  |  |  | Incubation time for virus is 14-28 days |
|  |  |  | Antibiotics are the first line treatment for the management of MERS-CoV |
|  |  |  | Polymerase chain reaction can be used to diagnose MERS-CoV |
|  |  |  | Washing hands vigorously (soap/water) for 20 seconds helps in prevention/transmission of disease |
|  |  |  | Vaccination of MERS-CoV is available in market |
|  |  |  | MERS-CoV is caused by alpha coronavirus |
|  |  |  | The main source of MERS virus is plant |
|  |  |  | Transmission of MERS-CoV infection can be prevented by using universal precautions. |
|  |  |  | MERS patients should be kept in isolation |
|  |  |  | Gowns, gloves, mask and goggles must be used when dealing with MERS patients |
|  |  |  | People with co-morbidities are more likely to be infected |
|  |  |  | Special caution must be taken if a person presents with MERS symptoms from the Arabian Peninsula |
|  |  |  | MERS-CoV patients develop severe acute respiratory illness |
|  |  |  | MERS-CoV spreads through close contact like caring and/or living with infected persons |
|  |  |  | Fever, cough and shortage of breath are hallmark symptoms of MERS-CoV |
|  |  |  | MERS-CoV can be fatal |

Kindly rate your perception to the following:

| **Strongly Disagree** | **Disagree** | **Agree** | **Strongly Agree** |  |
| --- | --- | --- | --- | --- |
|  |  |  |  | Prevalence of MERS can be reduced by active participation of healthcare workers in the hospital infection control program |
|  |  |  |  | Any related information about MERS should be disseminated among healthcare workers |

Thank you for your valuable participation
